# Prolonged abdominal pregnancy incidentally discovered during cesarean section: a case report

**DOI:** 10.1186/s12884-024-06358-6

**Published:** 2024-03-07

**Authors:** Hamidou Soumana Diaouga, Maimouna Chaibou Yacouba, Tidjani Mahamat Hissen, Maina Oumara, Inoussa Daouda Bako, Rahamatou Madeleine Garba, Nafiou Idi, Madi Nayama

**Affiliations:** 1 Department of Obstetrics and Gynecology, Abdou Moumouni University, Issaka Gazobi Maternity Hospital in Niamey, Niamey, Niger; 2 Department of Radiology, Abdou Moumouni University, General Reference Hospital, Niamey, Niger; 3 Department of Obstetrics Gynecology, Abdou Moumouni University, General Reference Hospital, Niamey, Niger; 4 Department of Obstetrics and Gynecology, Abdou Moumouni University, Maternity Unit of the Regional Hospital Center of Niamey, Niamey, Niger

**Keywords:** Abdominal pregnancy, Ectopic pregnancy, Ultrasound in the first trimester, Case report

## Abstract

**Background:**

Abdominal pregnancy is a rare medical condition that is still missed in developing countries due to inadequate medical facilities. The clinical indicators manifest in various forms and are nonspecific, making it challenging to diagnose and often leading to delayed detection. However, obstetric ultrasound serves as an essential tool in early detection. Our objective was to share our experience dealing with this condition and emphasise the importance of early ultrasound diagnosis through efficient pregnancy monitoring in our regions.

**Case presentation:**

35-year-old Black African woman who had ten months of amenorrhea sought consultation due to an absence of active foetal movements. Her pregnancy was of 39 weeks with fetal demise which was confirmed following clinical examination and ultrasound. She underwent cesarean section in view of transverse position of fetus. During cesarean section, the fetus was found within the abdominal cavity with the placenta attached over the left iliac fossa including surface of left ovary. The uterus and right adnexa were within normal limits. A 2600 g macerated fetus with placenta and membranes were extracted without any complications. The maternal outcome was successful.

**Conclusions:**

Abdominal pregnancy remained an inadequately diagnosed condition in developing countries. It is imperative to increase awareness among pregnant women regarding high-quality prenatal care, including early obstetric ultrasound, from conception. Meanwhile, healthcare professionals should receive continuous training and the technical platform modernised. To ensure accurate diagnosis, the location of the gestational sac must be identified for every pregnant woman during their initial ultrasound appointment.

## Introduction

Abdominal pregnancy is the implantation and development of a fertilised egg in the peritoneal cavity [1]. This form of ectopic pregnancy is rare and occurs with an incidence between 1/7000 and 1/15,000 deliveries in developed countries, although it is more prevalent in underdeveloped countries where medicalisation is inadequate, with an incidence between 1/1134 and 1/3750 deliveries [2–5]. The pathophysiology of this condition is still not well understood. The clinical signs are varied and non-specific, leading to a challenging and delayed diagnosis. Ultrasound is crucial for diagnosis [6]. Surgical intervention is necessary in almost all cases, and the prognosis for the foetus is typically poor. As for the mother’s prognosis, it is favourable when diagnosed at early gestation and modernisation of technical equipment [7]. We present a case of an intraoperatively discovered extremely prolonged abdominal pregnancy in a 35-year-old patient treated at our institute. The aim was to share our hospital’s experience where abdominal pregnancy remained undetected till advanced gestational age which emphasize the requirement for experienced sonologist for early ultrasound based diagnosis of this entity.

## Case presentation

**Fig. 1:**
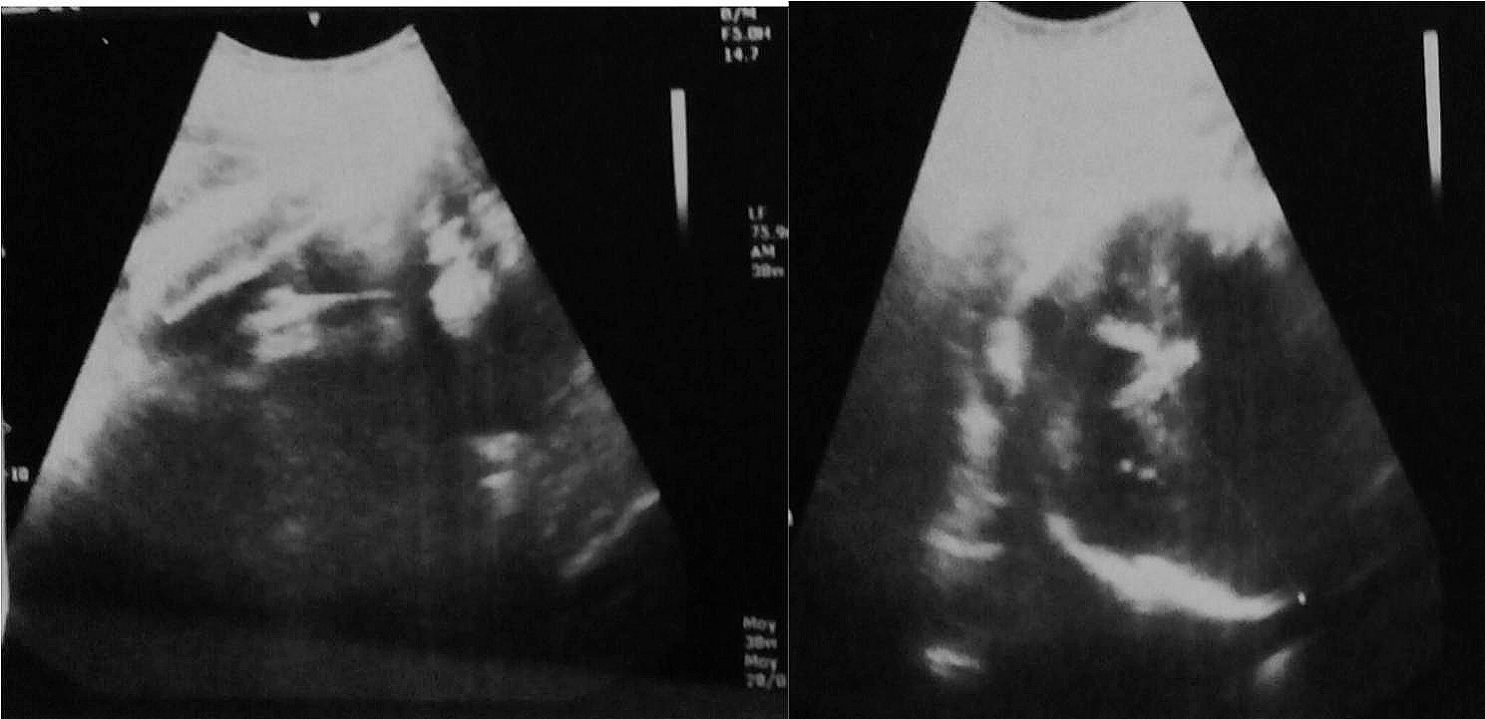
Obstetric ultrasound at 39SA. Note the absence of amniotic fluid and uterine structure circumscribing the fetal poles

**Fig. 2:**
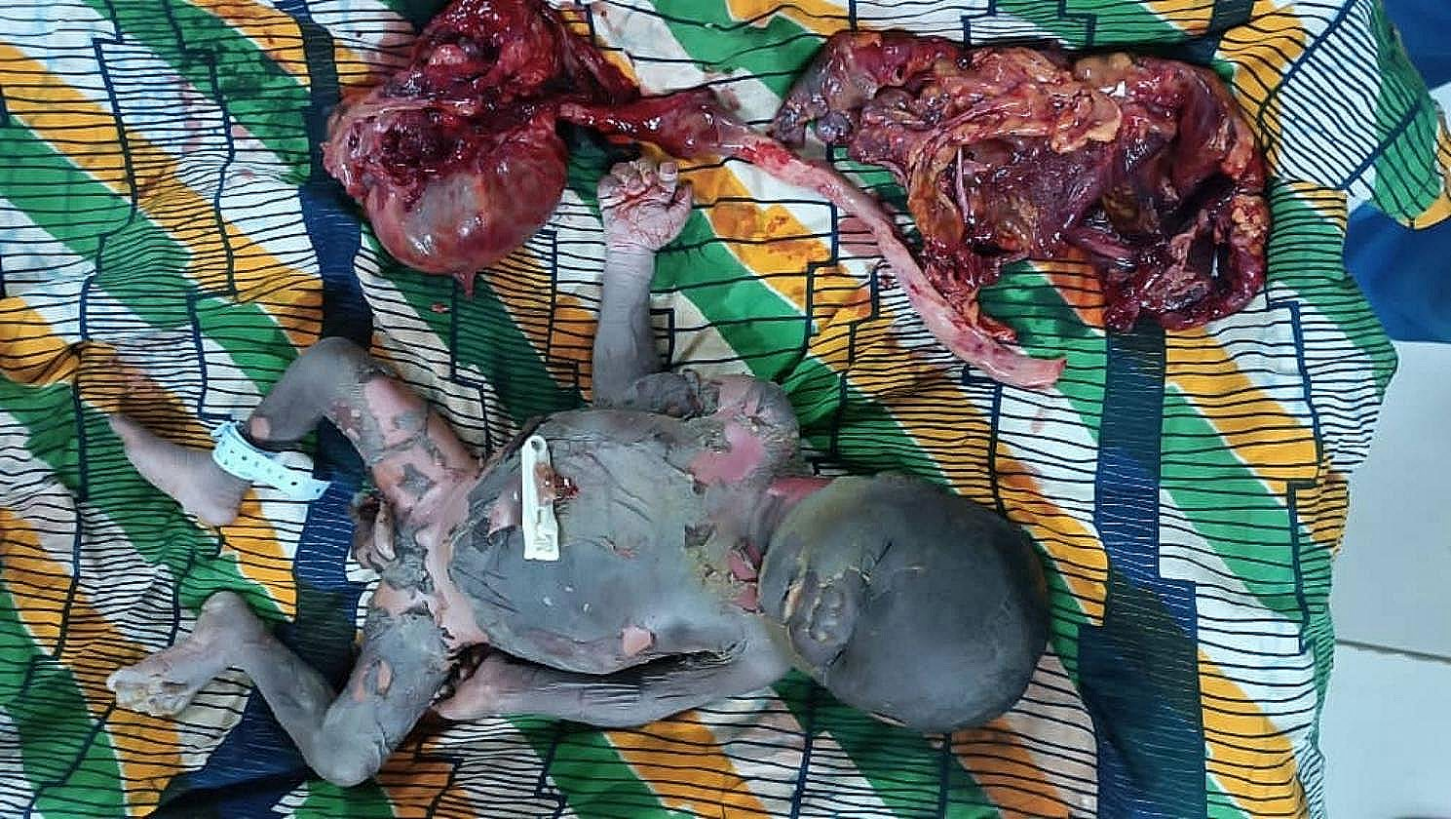
Operative parts of abdominal pregnancy. Note a mummified fetus, omentum, and left adnexa with placenta

It was a 35-year-old black African woman, gravida 6, anterior para 4 and mother of two living children. She had no prior history of comorbidity. On January 12, 2023, she sought medical care from our department for reduced fetal movements. As per the patient, she had been pregnant for over 10 months. A midwife had monitored her with five prenatal check-ups before admission, and she had never undergone any obstetric ultrasound. Throughout the first two trimesters of pregnancy, the patient reported experiencing chronic constipation and intermittent abdominal pain. However, there were no observed complications. The patient did not report severe pain or bleeding. Upon examination, the patient was deemed to be in good general condition, with normal conjunctiva and mucous membranes. Her blood pressure was recorded as 110/60mmHg. Obstetric examination revealed an irregular fundal height measuring 26 cm. The abdominal shape was irregular, causing pain during uterine mobilisation. Foetal heart sounds were not detected during auscultation. Upon performing a vaginal examination, it was observed that the cervix was long and posteriorly closed, and the pelvic cavity was empty. An obstetric ultrasound revealed a non-evolving monofetal pregnancy with overlapping skull bones, a transverse presentation of the foetus, and an absence of amniotic fluid. The foetal biometry was measured at 39 weeks (Fig. 1). The biochemistry panel revealed normal results. The search for disseminated intravascular coagulation returned normal findings. A transverse fetal presentation led to the diagnosis of fetal death, necessitating a caesarean section. Intraoperatively, the fetus was discovered within the peritoneal cavity with the placenta inserted into the left ovary.The uterus and right adnexa were normal. Numerous intraperitoneal adhesions were observed. Spinal anaesthesia was converted to general anaesthesia, and the originally performed horizontal incision was changed to a midline incision below and above the umbilicus, resulting in an inverted “T” incision. We performed extraction of a macerated foetus weighing 2600 g. Laborious adhesyolysis was performed and then a left adnexectomy removing the placenta. (Fig. 2) After checking that haemostasis was good, we rinsed the abdominal cavity with isotonic saline and closed the abdominal wall. A post-operative drain was placed in the peritoneal cavity.The postoperative period was uneventful.The patient was discharged on the 10th postoperative day in good health. On discharge the patient received the following advices: Consult very early in a maternity unit with ultrasound equipment for the next pregnancy, be regular in prenatal visits and do at least four ultrasounds, including two in the first trimester of pregnancy.

## Discussion

This report outlines a case of abdominal pregnancy in a 35-year-old woman who had given birth multiple times and was a homemaker with limited resources and education. Despite regular prenatal check-ups, the quality of these check-ups was inadequate as no ultrasounds were performed over the course of five consultations, leading to a missed opportunity for early diagnosis.

Abdominal pregnancy is a rare form of ectopic pregnancy that is exceptional in developed countries with an incidence between 1/7,000 and 1/15,000 deliveries. It is more common in underdeveloped countries and evaluated at 1/1134 in South Africa, 1/2583 in Senegal and 0.152% in Nigeria [2–5]. In Niger this rarity is not documented. The physiopathology is still poorly understood. The physiopathological mechanism makes it possible to distinguish secondary abdominal pregnancies, the most common, resulting either from a tubo-abdominal abortion, or from the rupture of a tubal ectopic pregnancy, or from the migration of an intrauterine pregnancy through ‘a breach of hysterotomy or uterine perforation. Rare cases of secondary abdominal pregnancies have also been described after hysterectomy [7]. Primary abdominal pregnancies are even rarer and are defined by Studdiford’s three criteria [2, 7] which are as follows: normal fallopian tubes and ovaries, absence of uteroperitoneal fistula and exclusive contact of the egg with the peritoneal surface. In our observation, it was a left ovarian pregnancy. The factors that might explain the higher prevalence of abdominal pregnancy in developing countries are: the sequelae of genital infections, often septic abortions, endo-uterine manoeuvres, and the lack of follow-up of pregnancies. In fact, in this case, a 6-week scan could have potentially prevented the issue. The literature highlights additional risk factors, such as in vitro fertilisation, uterine scarring, and intrauterine device contraception [2, 8]. No risk factors were identified in our patient. Diagnosis of abdominal pregnancy in clinical settings is dependent on the stage of the pregnancy. There are certain symptoms that indicate abdominal pregnancy [9]:

- Gastrointestinal problems such as nausea, vomiting, constipation, subocclusion,
- Abdominal and pelvic pain coinciding with fetal movements accompanied by or without metrorrhagia,
- Anaemia with poor overall health condition.
- A foetus in a superficial position, often in an atypical, high transverse position, was observed.
- A second pelvic mass corresponding to the enlarged but empty uterus was palpable.
- During vaginal examination, the cervix was often found to be fixed under the pubic symphysis, and it was hard and long.
- Sometimes, the presentation was complicated by the onset of various symptoms such as internal or external bleeding, anaemia, jaundice, oliguria, or a toxic-infectious syndrome.

Some of these signs were present in our patient.

Although the obstetric follow-up was conducted by a midwife, her presumptive signs were overlooked. It is important not to overlook the possibility of other serious causes, such as pancreatic cancer, despite this symptomatology. Pancreatic cancer is uncommon during pregnancy, but it can be severe and typically presents with symptoms such as abdominal pain, nausea/vomiting, weight loss and jaundice [10].

At the paraclinical level, ultrasound is crucial in diagnosing abdominal pregnancy. This is determined by the lack of parietal thickness between the maternal bladder and foetus, the proximity of fetal parts to the maternal abdominal wall, differing placental locations on ultrasound examinations, an ectopic placenta, abnormal fetus presentation, and the absence of amniotic fluid between the placenta and fetus [2, 6]. This patient only underwent one ultrasound at 39 weeks, prompted by her lack of fetal movement perception. She had to travel approximately one hundred kilometers to Niamey to undergo the ultrasound due to a shortage of obstetrician-gynecologists and radiologists in Niger. The ultrasound did not result in a diagnosis of abdominal pregnancy in our observation. This diagnostic process is easily comprehensible during the third trimester of pregnancy. Indeed, at this stage, the radiologist seldom requires information on the gestational sac’s position, which can be challenging to ascertain towards the end of the first trimester. In developed countries where magnetic resonance imaging (MRI) can be easily conducted, the diagnosis of certain conditions can be made more effectively. MRI can identify an empty uterus, a fetus in the abdominal cavity not surrounded by myometrial tissue, a frequently transverse presentation and oligo-amnios [3]. Therapeutically, the management of abdominal pregnancy typically involves surgical intervention through laparotomy, supplemented with methotrexate treatment if the placenta remains partially or fully in place. The therapeutic approach is dependent on the gestational age and necessitates a multidisciplinary team. Prior to 20 weeks, medical termination of pregnancy is frequently suggested [1, 5, 6]. After 20 weeks, the antenatal diagnosis of advanced abdominal pregnancy with a live foetus presents the challenge of deciding whether to continue or terminate the pregnancy. The primary issue during abdominal pregnancy procedures is the fate of the placenta, which is dependent on its insertion site. Due to the hemorrhagic nature of placental extraction, it is often necessary to leave it in place and perform a second intervention at a later time. The placenta atrophies, enabling its extraction with minimal blood loss. Our patient had successful extraction of the foetus and placenta in a single operation with good haemostasis. On the prognostic level, abdominal pregnancy is an obstetric emergency. This condition poses a threat to the individual’s life due to the possibility of rupture, leading to massive haemorrhage. The first trimester and early second trimester of pregnancy are more susceptible to complications. Foetal prognosis is bleak, with a high mortality rate between 75 and 95% [5, 7, 9]. Causes of death are associated with foetal hypotrophy and malformations. These factors, linked to inadequate vascularisation of the placenta, potentially account for the foetus’s fragility. The maternal prognosis is reliant on the early detection of the issue and the technical capabilities of the care team. Maternal fatalities can range from 0 to 18%, typically caused by bleeding and infectious complications [7, 11]. Our patient progressed favourably.

## Conclusion

Abdominal pregnancy is a rare pathology in developed countries. However, it is more common in underdeveloped countries, where it often occurs in advanced stages due to inadequate medical care and low socio-economic status. Diagnosis and treatment are frequently challenging in our setting. Despite advancements in anaesthesia-resuscitation, the maternal and foetal outlook is still bleak, particularly in deprived and under-resourced areas. It is essential to increase awareness among pregnant women regarding high-quality prenatal care, starting from conception. This can be achieved by the continuous training of health professionals and modernizing the technical platform. The location of the gestational sac should be diagnosed in every pregnant woman during their first ultrasound appointment. During a subsequent pregnancy, it is imperative to be under the care of a specialist at a centre equipped with ultrasound facilities.

## Data Availability

Not applicable.
